# Trajectory maps: molecular dynamics visualization and analysis

**DOI:** 10.1093/nargab/lqad114

**Published:** 2024-01-15

**Authors:** Matej Kožić, Branimir Bertoša

**Affiliations:** Department of Chemistry, Faculty of Science, University of Zagreb, Horvatovac 102a, HR-10000 Zagreb, Croatia; Department of Chemistry, Faculty of Science, University of Zagreb, Horvatovac 102a, HR-10000 Zagreb, Croatia

## Abstract

Molecular dynamics simulations generate trajectories that depict system's evolution in time and are analyzed visually and quantitatively. Commonly conducted analyses include RMSD, *R*_gyr_, RMSF, and more. However, those methods are all limited by their strictly statistical nature. Here we present trajectory maps, a novel method to analyze and visualize protein simulation courses intuitively and conclusively. By plotting protein's backbone movements during the simulation as a heatmap, trajectory maps provide new tools to directly visualize protein behavior over time, compare multiple simulations, and complement established methods. A user-friendly Python application developed for this purpose is presented, alongside detailed documentation for easy usage and implementation. The method's validation is demonstrated on three case studies. Considering its benefits, trajectory maps are expected to adopt broad application in obtaining and communicating meaningful results of protein molecular dynamics simulations in many associated fields such as biochemistry, structural biology, pharmaceutical research *etc*.

## Graphical Abstract

**Graphical Abstract:**
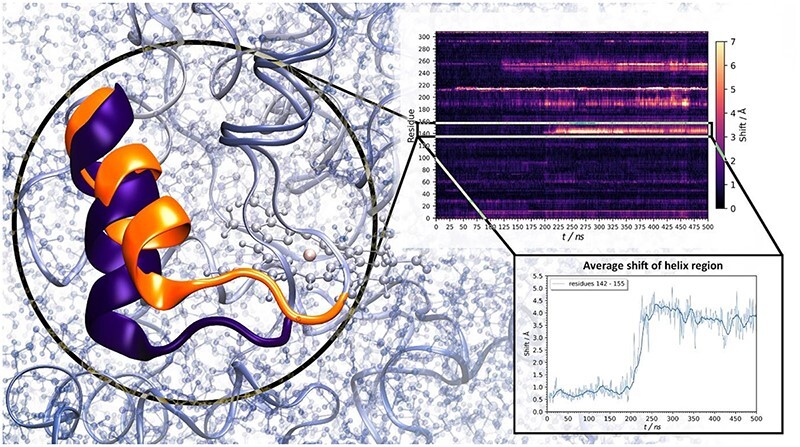

## Introduction

Molecular dynamics (MD) simulations of proteins are an invaluable tool in many branches of life sciences, from biophysics, biochemistry, structural biology, to molecular biology and more. With uses in academia as well as industry, some noted applications include drug discovery in pharmaceutical sciences (1), discovering and creating improved protein variants (2), gaining biological insights (3), *etc*. To gather the most insight from simulated systems, various methods of analyzing MD simulations of proteins have been developed over the years. Visual analysis of the simulated system is conducted by observing the simulation throughout its course, and then complemented with quantitative analyses. Some of the most common statistical analyses include root mean square deviation (RMSD), radius of gyration (*R*_gyr_), root mean square fluctuations (RMSF), and more. Given the complexity of simulated systems robust, fast, and easy to interpret methods of analyses are of great value and significance to both researchers conducting them and the audience interpreting them. Intuitive and unambiguous visualizations of quantitative analyses can simplify the distinction of important results from the ever-present large data noise, minimize human error, and facilitate communication of information and scientific discourse of research findings.

Presented here is a novel method of representing protein molecular dynamics simulations, complex multidimensional systems, as two-dimensional heatmaps of proteins’ backbone movements. This approach, referred to as trajectory maps, offers intuitive visualizing of simulation courses, direct conclusive comparison of multiple simulation courses, plotting movements of specific regions of proteins during the simulation and more.

## Materials and methods

### Trajectory maps

The foundation of trajectory maps is in movements of amino-acid residues from their reference positions, referred to as shifts. Shifts, defined as a Euclidean distance of centers of masses of residues backbone in time ***t*** from reference time ***t_ref_***, are shown in a matrix for every residue and every frame of the simulation. The expression used for calculating shifts is shown with equation 1.


(1) \begin{eqnarray*}s\left( {r,t,{t}_{ref}} \right) = \sqrt {{{\left( {{x}_{r,t} - {x}_{r,{t}_{ref}}} \right)}}^2 + {{\left( {{y}_{r,t} - {y}_{r,{t}_{ref}}} \right)}}^2 + {{\left( {{z}_{r,t} - {z}_{r,{t}_{ref}}} \right)}}^2}\nonumber\\ \end{eqnarray*}

A shift ***s*** is calculated for every residue ***r*** in time ***t*** against reference time ***t_ref_*** which is taken as the first frame of the simulation. Coordinates *x*, *y* and *z* are of the center of mass of a residue's backbone atoms: Cα, C, O and N. A matrix of shifts is created and its values are color coded. The resulting heatmap that represents the MD trajectories is in the text referred to as a trajectory map.

Before subjecting trajectory to the trajectory maps analysis, frames have to be aligned so that rotation and/or translation of the whole systems is extracted. This can be achieved either in the simulation setup or in the trajectory processing by alignment of frames using *trjconv* command in GROMACS or *align* command in AMBER. Also, optimal performance of trajectory maps is achieved on trajectories containing between 500 and 1000 frames. So, the reduction of number of frames from original trajectory is recommended in order to obtain the most clear, readable, and easy to interpret trajectory map.

In given examples shifts were calculated using the center of mass of the backbone atoms, but any other points could be used instead e.g. only the positions of α-carbon atoms of the backbone or the center of mass of the whole amino acid residues, etc. Using centers of mass (as opposed to Cα positions) results in a better resolution of the map's *z* axis (representing shifts) because in-residue vibrations are diminished in magnitude. Furthermore, a reference from which shifts are calculated is taken as the first frame of the simulation, *t*_0_, which can also be modified. By taking the previous timestep as a reference (shift from *t*_*i-*1_ to *t_i_*) a map can be obtained as well. A map of ‘previous step’ shifts is independent of conformational changes and only shows the amount of fluctuations in the protein. Since it is dependent on the previous step of trajectories, the choice of trajectory stride is critical. At the moment, this feature is deemed less useful than having the reference time be the first step of a simulation. For the reasons discussed none of these two features are available in the main Python program herby provided, but can be accessed by manually modifying the source code.

While there exist in literature several instances of approaches similar to this, notably hereby mentioned references (4–6) and a feature in a plugin of a program VMD (7), the authors state that this is the effort of an original idea developed independently and solely by the stated authors. Furthermore, regarding literature, to the best of author's knowledge none have neither performed nor utilized it neither in a way nor to an extent as described per this article.

### Implementation of trajectory maps

Implementation of trajectory maps can be achieved through TrajMap.py, an easy-to-use open-source Python-based script. TrajMap.py (TM) is dependent on four Python libraries: Numpy (8), Pandas (9), Matplotlib (10), and MDTraj (11); which can all be easily installed using *pip*. The script is ready to be used as-is, and on Windows operating systems it is recommended to be used through Anaconda distribution of Python. On Linux operating systems, TM can be used in two ways: (i) as a terminal application by manually entering inputs and (ii) with Bash scripts with pre-written inputs. Usage through Bash scripts is recommended as it is faster and easier than manual typing, and allows for upscaling and bulk processing, mitigating the room for human error. Both ways of usage require virtually no knowledge of Python or Bash, and user-friendly guidelines are present in both approaches.

Features of TM include: (i) creating a trajectory map from a simulation, (ii) creating a shift-graph of a defined region of a protein, (iii) calculating an average of two or three trajectory maps and (iv) calculating the difference of two trajectory maps (of singular simulations, or simulation averages).

**Figure 1.**
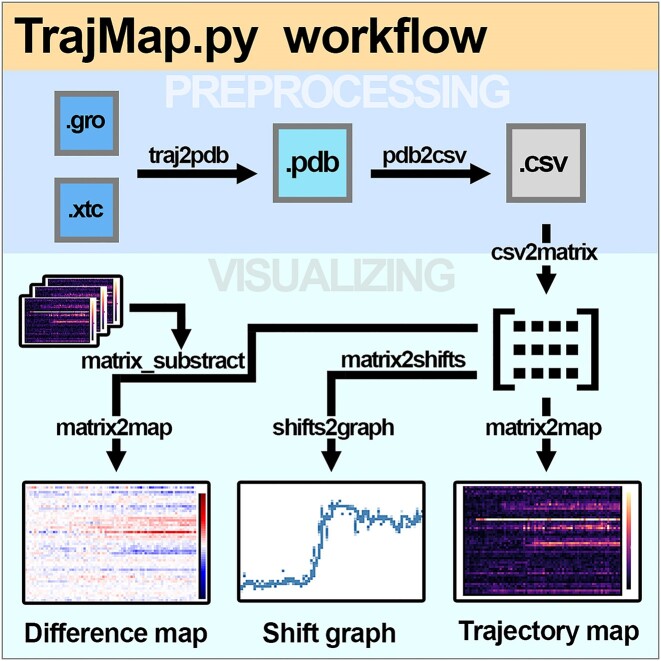
A schematic representation of TrajMap.py Python programming language terminal program/a Python library for trajectory map analysis. For a detailed explanation, refer to the documentation.

The workflow centers around converting trajectories and topologies (.xtc + .gro; .xtc + .pdb; .nc + .prmtop; *etc*.) into a matrix of shifts that is saved as a .csv file. That constitutes the first step: preprocessing. In the second: making the map; the .csv matrix is loaded, and a map is created from it using inputted parameters. The reason for this two-step approach is that preprocessing is the longest step, taking around 5 min for 300 residues 500 frame simulation; and creating the map is significantly faster but could require multiple iterations to fine-tune the range of the *z* axis color-scale, and/or axes ticks, *etc*. The workflow scheme is provided with Figure 1.

TrajMap.py comes as TrajMap kit, with the main script TM.py, premade Bash scripts (preprocessing script and map-making script), and a ‘test kit’ folder. In it are included small (under 50 MB) test trajectories and a Bash script that runs the program and all features with test trajectories to make sure all modules are imported and working properly. Extensive documentation is provided as well, with detailed instructions on how to install the dependencies and use the module. Alongside the TM program TrajMap_local library is provided as well, and it can be used as a locally imported Python module. The full access to functions is therefore available, and they can be used as from any other a Python library. With sufficient knowledge of Python, axes names, figure sizes, colormaps, reference times and everything else can be modified and changed.

## Results

### Trajectory maps

Trajectory maps show the residue's backbone shift (defined as an Euclidean distance) from a residue's backbone's starting position of the simulation, in each frame of a simulation. On the *x* axis are the frames of the trajectories (simulation time), on the *y* axis are the residues (residue number), and on the *z* axis is the color scale representing the magnitude of a shift in each frame from the backbone's position in the first frame. Consequently, trajectory maps show the location, time, and magnitude of every movement of a protein's backbone during the simulation. This intuitive visualization of the full course of a simulation can greatly aid in understanding and interpreting it, especially in the early stages of analysis. Having a roadmap of conformational events can facilitate and speed up the visual analysis of studied trajectories, as well as indicate the future courses of analyses. Furthermore, it allows for comparing multiple simulations in a conclusive and intuitive way. Validation the of the method is provided through three case studies where trajectory maps were applied to study already investigated systems.

### Case study 1: differentiating stability of simulated systems

**Figure 2.**
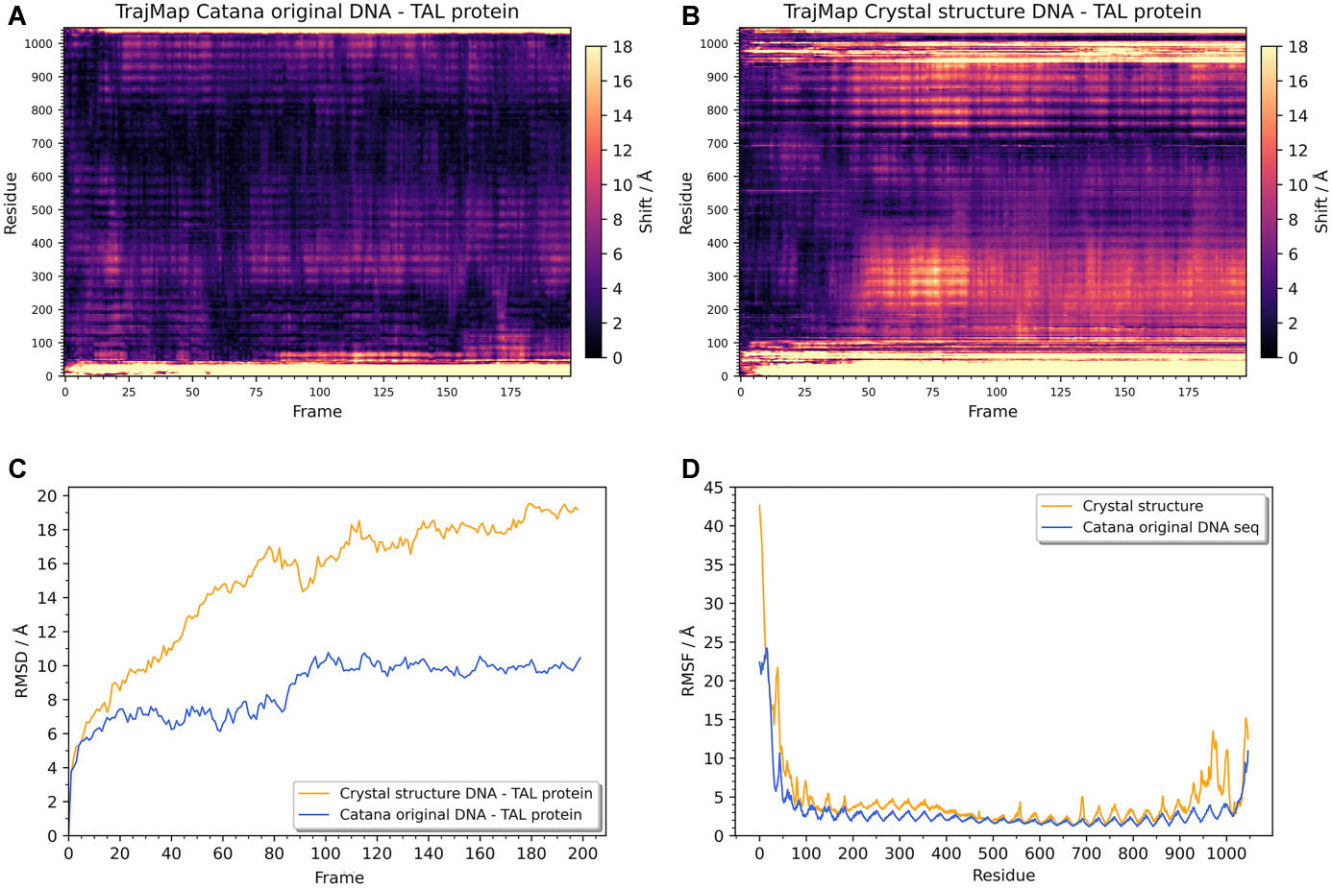
Analysis of molecular dynamics simulations of a TAL protein in complex with DNA. Panels **A** and **B** show trajectory maps of two individual simulations, and panels **C** and **D** show RMSD and RMSF graphs respectively. Protein-DNA complexes were generated from scratch using CATANA (panel **A** and blue lines on panels **C** and **D**) or prepared starting from a crystal structure (panel **B** and yellow line on panels **C** and **D**).

Authors of a study titled ‘CATANA: an online modelling environment for proteins and nucleic acid nanostructures’ (12), tested their modelling tool by comparing simulations starting from structures built with it, against structures obtained by other common means (usually starting from the crystal structure). One of the simulated proteins, Transcription activator-like effector (TAL) (13), was simulated in a complex with a DNA sequence built with CATANA, and compared against a simulation of a TAL complex built with a crystal structure DNA sequence (Figures 2A and B).

Trajectory maps for both simulations when compared show the difference in stability, indicating that a TAL complex with CATANA-built DNA is more stable. Those results are further confirmed by RMSD and RMSF graphs, two commonly used analyses (Figure 2B and C). Compared to RMSD and RMSF graphs, trajectory maps reveal additional detail in the form of starting times, locations, and magnitudes of even temporary conformational events. Conclusions drawn from trajectory maps are in accordance with the authors’ conclusion that CATANA structure built using Alphafold leads to a more stable protein complex compared to a complex built from crystal structure using other homology modelling tool (4). Additionally, trajectory maps reveal the regions of most instability and time frame in which they happen. Insights gathered in that way can be further explored through other means such as visual analysis.

### Case study 2: comparing structural dynamics of multiple systems

Multiple simulations can easily be compared by subtracting their trajectory maps (e.g. A–B for simulations A and B). Shifts stronger in A yield positive values, and shifts stronger in B yield negative values, while similar shifts stay close to zero. Coloring the resulting difference map with a colormap divergent around a value (e.g. blue-white-red) makes positive values (shifts stronger in A) red, regions of similar shifts white, and regions with shifts stronger in B blue. Additionally, multiple simulations can be averaged out and the averages can be subtracted (e.g. subtracting the average of duplicates A and B: [A + A′ – B – B′] / 2).

From trajectory maps shift graphs can be created by plotting the shift in time of a single residue as a two-dimensional graph. Same can be done for trajectory difference maps as well. Furthermore, an average of a region can be plotted to show the shift in time of a portion of a protein (e.g. a whole helix, a whole domain, *etc*.). That is analogous to looking at a desired chunk of a trajectory map through the *y* axis, so the trajectory map color scale *z* axis becomes the *y* axis of a newly constructed graph.

**Figure 3.**
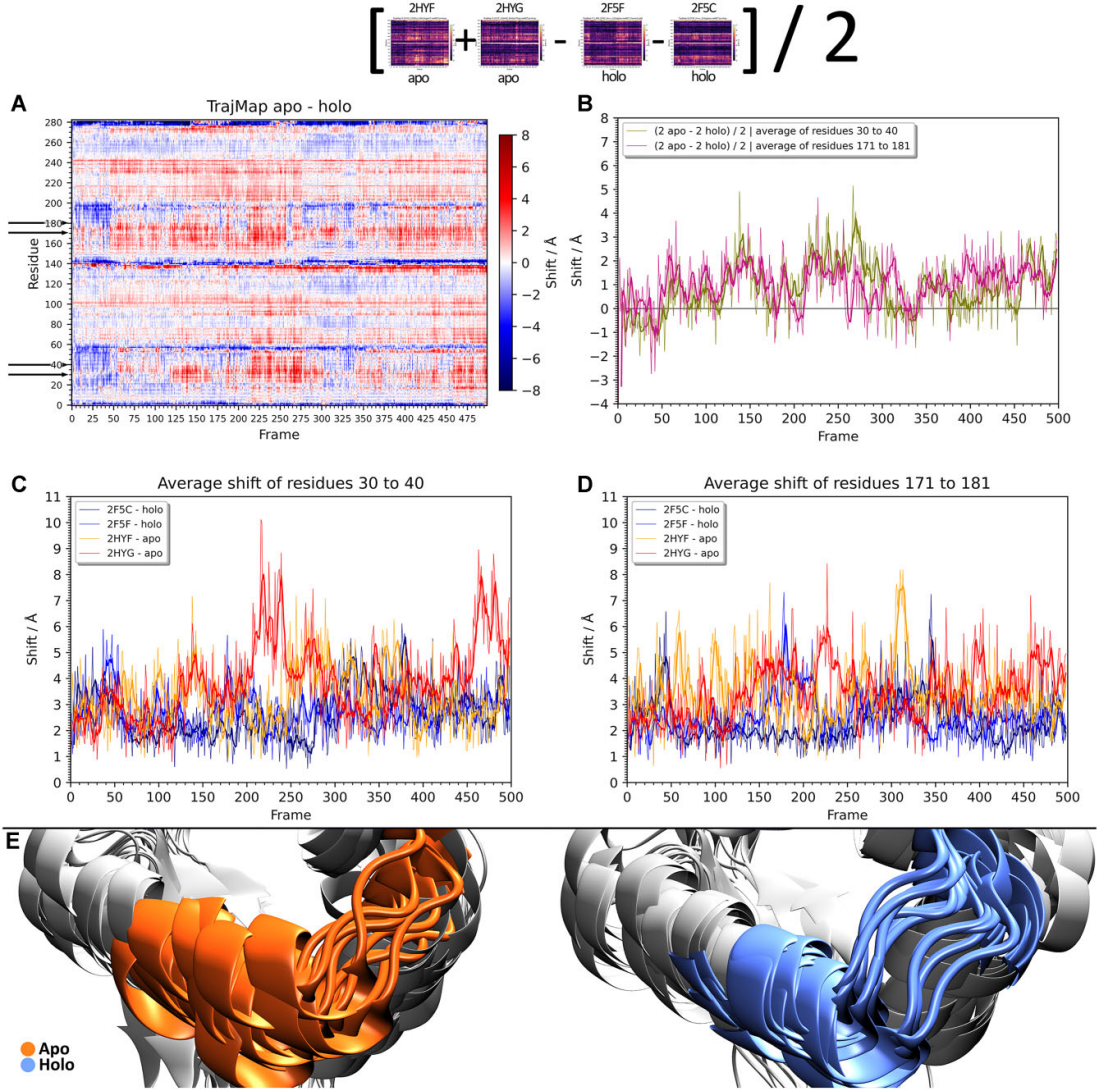
A detailed analysis of a system of four simulations with trajectory maps. On the top, a schematic representation of creating a trajectory difference map of averages of apo and holo forms, shown in (**A**). Red (positive) pixels represent areas and times where shifts were bigger in apo form. Blue (negative) regions represent areas and times where shifts were bigger in holo form. Whites (zeroes) represent similar magnitude of shifts in both apo and holo form. (**B**) A plot of average shifts of residues 30–40 (green) and 171–181 (purple) of the difference map (shown with black arrows on the *y* axis of the difference map). (**C**) A plot of average shifts of the region 30–40 of two apo (red and orange) and two holo (blue and navy) forms, obtained from trajectory maps of individual respective simulations. (**D**) A plot of average shifts of the region 171–181 of two apo (red and orange) and two holo (blue and navy) forms, obtained from trajectory maps of individual respective simulations. On graphs (**B**–**D**) is superposed a moving average of 10 frames. **E**) Region 30–40 of one apo (orange) and one holo (blue) simulation shown as a superposition of snapshots taken every 50 ns of the 500 ns MD simulation.

In a study titled ‘Structural dynamics of the *Bacillus subtilis* MntR transcription factor is locked by Mn^2+^ binding’ (14), Jelić Matoševć *et al.* studied the role of manganese ions in structural dynamics of a MntR protein, a homodimeric transcriptional factor from *Bacilus subtilis*. From that study, two simulations of a holo protein (in a complex with Mn^2+^ ions) (in the referenced article, structures with PDB codes 2F5F and 2F5C) (15) and two simulations of an apo protein (in the referenced article, structures with PDB codes 2HYG and 2HYF) (16) were used for testing trajectory maps. From the four simulations, trajectory maps were generated, and by subtracting the average of holo simulations from the average of apo simulations, the differences between the systems were shown (Figure 3A). From there, each band representing a difference between simulations can be described in the context of the protein. Blue regions represent shifts stronger in the case of the holo form of the protein, while red regions represent shifts stronger in the apo form. White indicated shifts that were of the same magnitude in both forms. A region identified as of importance was a DNA binding region that includes residues 30–40 and 171–181 (black arrows on the *y* axis of Figure 3A) (those are the same chain regions because homodimeric chains were numerated 1–141 and 142–282). The average shift of a regions 30–40 and 171–181 were plotted for the difference map of averages (Figure 3B) and for individual simulations (Figure 3C and E).

The trajectory difference map shows how for regions 30–40 and 171–181 shifts are stronger in the case of apo form. That is quantified by panels B–D, which all confirm that respective regions lose stability in holo form. Additionally, the four-simulation difference of shifts (panel B) demonstrates similar behavior of both residues 30–40 and 171–181, which is according to expectations as those are the same regions of two homodimeric chains. The conclusion is that holo forms, with a bound manganese ions, have the mentioned regions more stable and with less fluctuations than in the apo forms where the fluctuations are more pronounced. The quantified results are visually shown in the panel E, where it is visible how the orange apo form has more disordered fluctuations that the blue holo form; although that conclusion is less clear than shifts graphs. Conclusions drawn from trajectory map analysis support the author's conclusion that the binding of Mn^2+^ ions reduces the conformational space of the protein and locks the orientation of the DNA-binding helices (14). Results obtained with trajectory maps are further confirmed by author's PCA analysis of proteins’ backbones, and other means utilized in the original article (which can be further quantified and given intuition and context by trajectory map analysis). Again, the same conclusions were obtained by the authors, but with much more effort in visualization and usage of variety of different analyzing tools. On the other hand, the trajectory difference map directly pointed out these regions and would have significantly reduced the effort and the time required from the authors to obtain and convey same results (if it were available at the time of the study).

**Figure 4.**
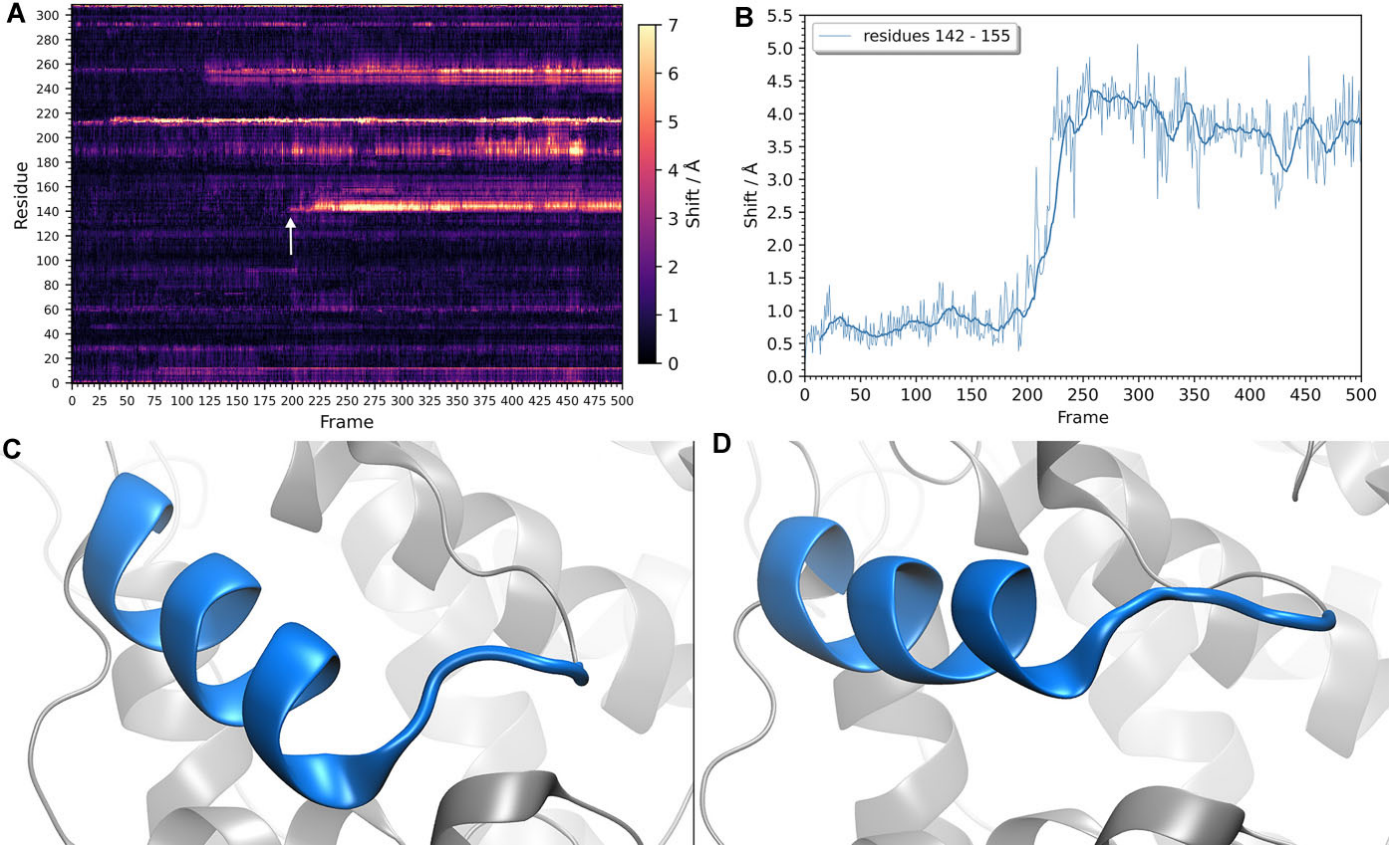
A trajectory map analysis of a recombinant type of horseradish peroxidase enzyme MD simulation. (**A**) A trajectory map of the simulation; the white arrow indicates the band at region 142–155 whose shifts are plotted on a shift graph on the panel **B**. Region 142–155 (shown in blue) before the conformational change, on panel **C**, and after the conformational change, on panel **D**. On the shift graph is superposed a moving average of 15 frames.

Additionally, shift graphs reveal that the observed effects alter only the fluctuations of the protein structure without causing any significant conformational change, the same was concluded by the authors of the study (14). In case of a stable conformational change, the shifts would increase and stay fluctuating at some values around a new stable conformation; which is shown in Figure 4 in the following section Case Study 3 where it is a case of a change in a conformation and not only an increase in fluctuations.

### Case study 3: quantifying conformational changes

In an ongoing study, a variant of an enzyme horseradish peroxidase (HRP) (17) with several known mutations (a recombinant type) is being studied. The recombinant type of HRP (mHRP) was simulated with classical molecular dynamics. Visual analysis showed a change in a helix region of the enzyme near the active site (residues 142–155, Helix E). A trajectory map was generated, and a notable band corresponding to that region confirmed the magnitude of that change (Figure 4A, white arrow). A shift graph of that region was plotted, thereby quantifying the results (Figure 4B). Furthermore, the trajectory map showed the existence of two more notable bands responding to conformational changes near the helix E region (on the trajectory map, band at residues ∼250), which later in the study aided the characterization of the mechanism by which it happens.

**Figure 5.**
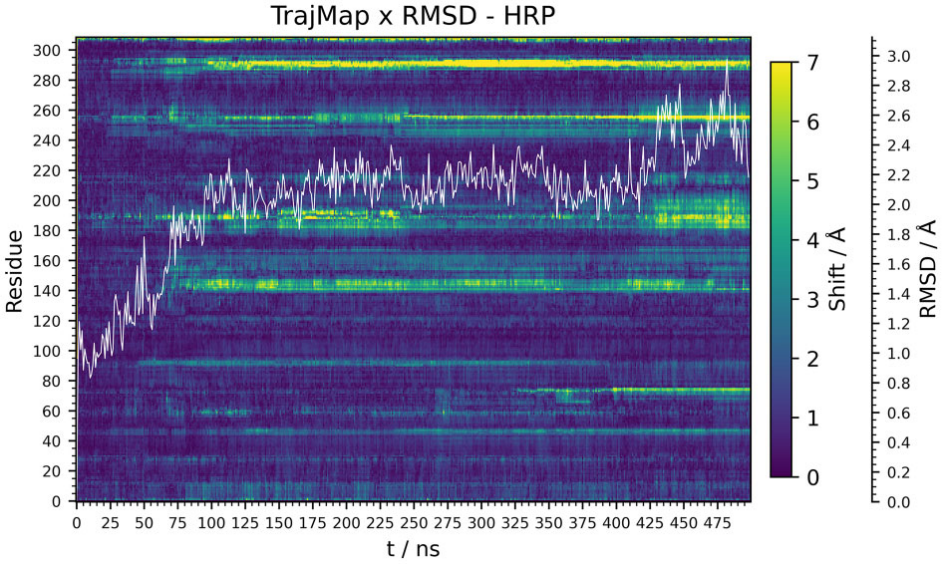
A trajectory map of a HRP simulation with a superposed RMSD graph of Cα atoms of protein's backbone (shown as white line).

In another instance of a mHRP simulation the mentioned conformational change was once again observed. To show its impact on the structure of HRP, a trajectory map was created and on it was superposed an RMSD graph (Figure 5). A jump in an RMSD graph in time frame at which is observed the start of helix E conformational change confirms and quantifies its overall impact on the structure. With an RMSD graph superposed on the map, the impact of individual conformational events on the overall stability of the enzyme can be seen and quantified, as shown in this example.

**Figure 6.**
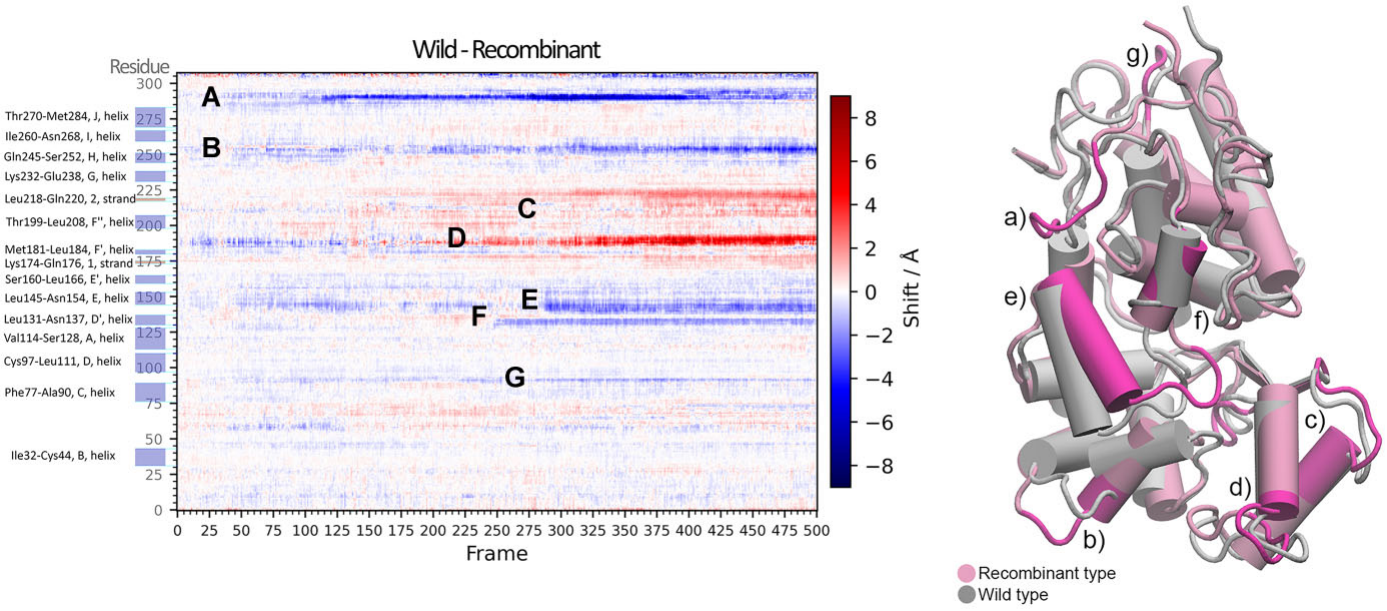
A difference map of average of four increasing temperatures of a wild type enzyme horseradish peroxidase (HRP) from a recombinant type HRP with several mutations. Positive (red) regions respond to shifts being stronger in the case wild type, and blue (negative) regions respond to shifts being stronger in the recombinant type. White regions represent shifts of the similar magnitude that cancelled out to zero. On the *y* axis are annotated secondary structures from literature, and on the map notable bands are assigned letters (**A**–**G**) used for characterizing and referencing them on the enzyme. On the right, a single frame horseradish peroxidase simulation of a wild type (gray) superposed on a single frame of a recombinant type simulation (pink); with a cartoon model of cylinders representing helices. Notable bands (**A**–**G**) from the trajectory difference map are assigned their structural position and highlighted with a more intensive pink.

Further in the study, HRP was simulated at four increasing temperatures in a range from 300 K to 353 K, for both the recombinant and the wild type (resulting in two batches of four simulations). Trajectory maps were generated, and an average across four temperatures for the recombinant type was subtracted from the temperature average of the wild type. The resulting difference map (Figure 6) showed all the difference of conformational events of backbones in wild and recombinant type, adjusted for increasing temperatures. To aid the characterization, secondary structures from literature were annotated on the *y* axis. From there it was easy to assign and characterize individually on the enzyme each band that responded to a difference between simulations (negative values showed shifts stronger in the recombinant type while positive values showed shifts stronger in wild type). From that, the effects of mutations on the structural dynamical properties of the enzyme, as related to an increase in temperature, were studied and interpreted. This constituted a fully conclusive overview and comparison of backbones’ conformational events of two batches of four simulations (eight simulations in total).

## Discussion

Trajectory maps, a novel method of visualizing and analyzing protein molecular dynamics simulations, was presented. With trajectory maps it is possible: (i) to visualize the full course of a simulation in an elegant and intuitive way, (ii) to directly compare courses of multiple simulations or multiple simulation sets, (iii) to visualize the behavior of a residue or a protein region during the simulation, (iv) to distinguish between significant conformational changes or increases in fluctuations and (v) to complement the established methods of analyses, such as RMSD and RMSF. The elegant and intuitive manner in which trajectory maps convey the full course of a simulation, handle multiple variant simulations, and visualize shifts of regions (in addition to other tools arising from this approach), could greatly benefit many fields associated with protein molecular dynamics simulations. Several of the benefits researchers could have from applying trajectory maps for analyzing molecular dynamics trajectories were demonstrated on three previously investigated systems.

Backbone movements and conformational changes of regions are shown as a horizontal band on a heatmap that represents trajectories. Distinguishing start times of individual conformational changes as bands on a heatmap can aid in interpreting the results of other analyses such as RMSD, *R*_gyr_ and others. By comparing an RMSD graph with a trajectory map it is possible to assign RMSD peaks with individual conformational changes that caused them. Trajectory maps provide a way to distinguish between conformational changes that lead to a stable different conformation of a region, and destabilizing changes that result in an increase in fluctuations; which complements the RMSF analysis (where this distinction can be ambiguous). Furthermore, a useful feature of trajectory maps is their ability to showcase the order in which conformational changes occur. By examining the starting times of bands responding to shifts of regions involved in a mechanism, it is simple to deduce the order in which these changes occur; which can aid in characterizing a mechanism of that conformational change.

Another powerful feature is its ability to plot the average shift of a defined region throughout time, in the form of a 2D shift-graph. The resulting shift-graph can be used to show the changing position of structural elements e.g. a helix throughout the simulation, as a proof of a conformational change/proposed mechanism. This is analogous to looking at a defined slice of a trajectory map through the *y* axis, so the color-coded *z* axis responding to the magnitude of a shift becomes the *y* axis of the constructed graph.

Since heatmaps are color-coded matrices, it is possible to perform calculations with them. Several heatmaps can be averaged out to show the average course of e.g. a triplicate simulation. By subtracting two matrices (e.g. *apo – holo*, *recombinant – wild, etc*.), all the differences of their courses and conformational changes are concisely and conclusively shown. The resulting trajectory difference map is color-coded with a divergent colormap to highlight the differences (divergent colormap diverge around a value in both positive and negative values, e.g. blues for negative values, whites for zero values, and reds for positive values).

TrajMap.py, an easy-to-use open-source Python-based script created for generating trajectory maps, was provided, together with clear and simple instructions for its usage. Provided content includes the main script, Bash scripts for easy and fast usage on a larger scale, and documentation with detailed descriptions of its workings.

Trajectory maps is a simulation analysis tool that is simple to use and provides the results that are easy to comprehend and interpret. In order to demonstrate advantages of trajectory maps over similar tools, Case studies 1 and 2 were also analysed by other tools and comparison of the obtained results is provided in Supplementary Data (Supplementary Figures S1–S4 in Supplementary Data). The advantage of trajectory maps over similar tools in the presentation of the obtained results can be seen for both cases. Furthermore, the required skills to obtain and to present such results (Supplementary Figures S2a, S2b and S4b in Supplementary Data) are significantly more demanding in case of other similar trajectory analysis tools than in the case of trajectory maps. In addition, it is worth noting that such usage, as is presented in our case for comparison purposes, isn’t mentioned in the documentation of tools we compared to trajectory maps, and to the best of our knowledge hasn’t been previously performed in that way in any literature. The best example of the impact that trajectory maps could have in the field of MD simulations is seen from the Case study 3. In that case, subtle conformational changes during eight simulations were identified and, more importantly, impact of each conformational change on the overall stability of the enzyme was quantified. Without trajectory maps the same analysis would be likely impossible. With the insight we present in this paper, it might be possible, but it would require deep understanding of the mathematical background of this and similar methods of analysis, as well as an advanced usage of tools we compared to trajectory maps. For sure, it would be demanding and time consuming. Therefore, trajectory maps present opportunity for even a beginner in the field to perform such comprehensive and powerful analyses in a simple and straight forward way.

## Supplementary Material

lqad114_Supplemental_File

## Data Availability

The source code for TrajMap.py is open source and fully available on Zenodo at DOI: 10.5281/zenodo.10428488 (https://zenodo.org/doi/10.5281/zenodo.10428488). The Zenodo repository serves as a permanent backup of the original GitHub repository cited in this publication (https://github.com/matkozic/TrajMap) with a permanent DOI.
