# Supplementary material for: Trajectory maps: molecular dynamics visualization and analysis
Source: NAR Genom Bioinform. 2024 Jan 15;6(1):lqad114. doi: 10.1093/nargab/lqad114 (PMC10789246; doi:10.1093/nargab/lqad114)
Supplement: lqad114_Supplemental_File [file lqad114_supplemental_file.docx]

**Supplementary Data**

**Trajectory maps: molecular dynamics visualization and analysis**

Matej Kožić^1^ and Branimir Bertoša^1,^*

^1^ Department of Chemistry, Faculty of Science, University of Zagreb, Horvatovac 102a, HR-10000 Zagreb, Croatia

* Correspondence: Branimir Bertoša, Tel.: +385 1 4606 132; Fax: +385 1 4606 131; Email: bbertosa@chem.pmf.hr


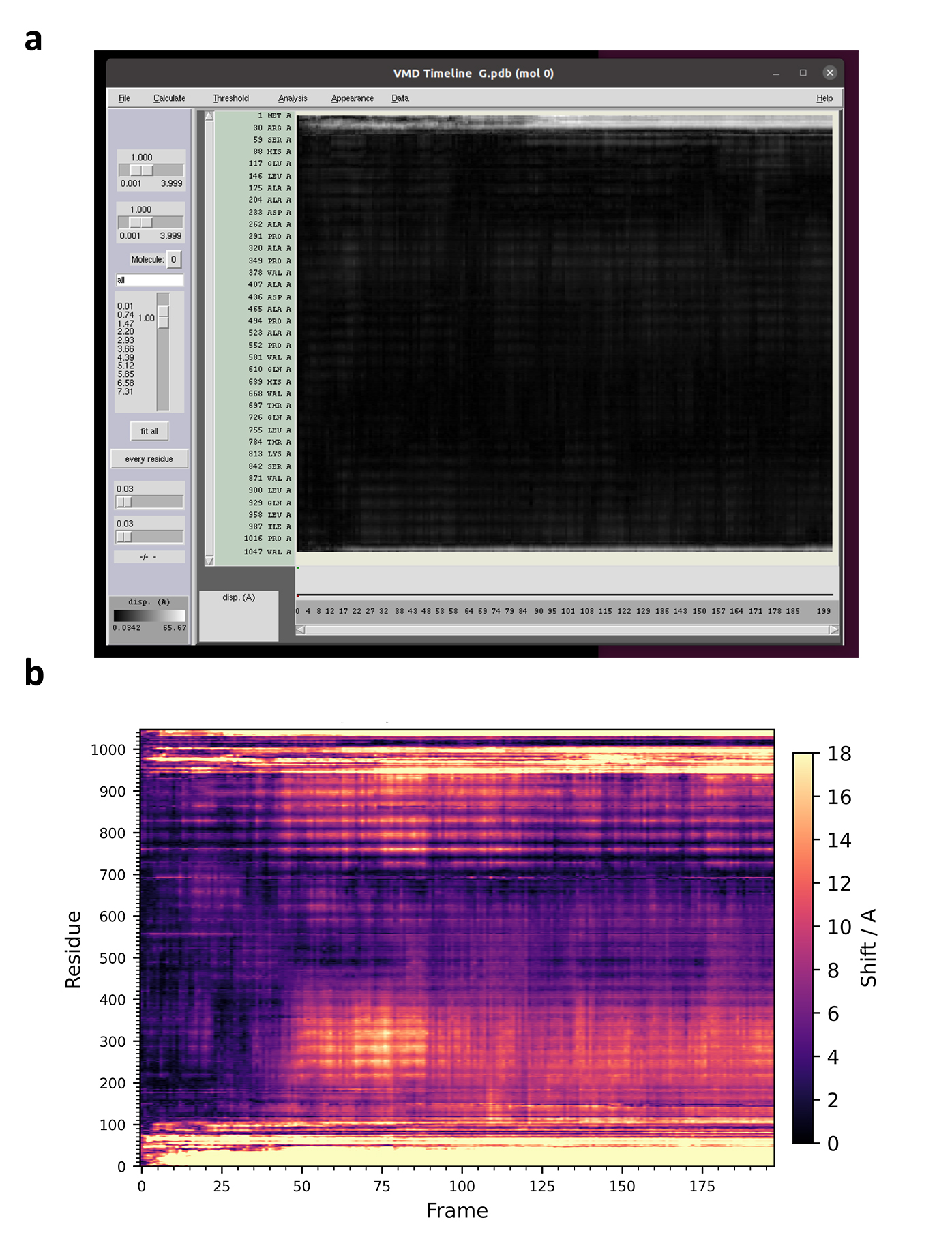


**Figure S1.** Comparison of: a) default view of VMD Timeline tool and b) heatmap obtained by trajectory maps of the same molecular dynamic simulation of the TAL complex built with a crystal structure DNA sequence (Case study 1).


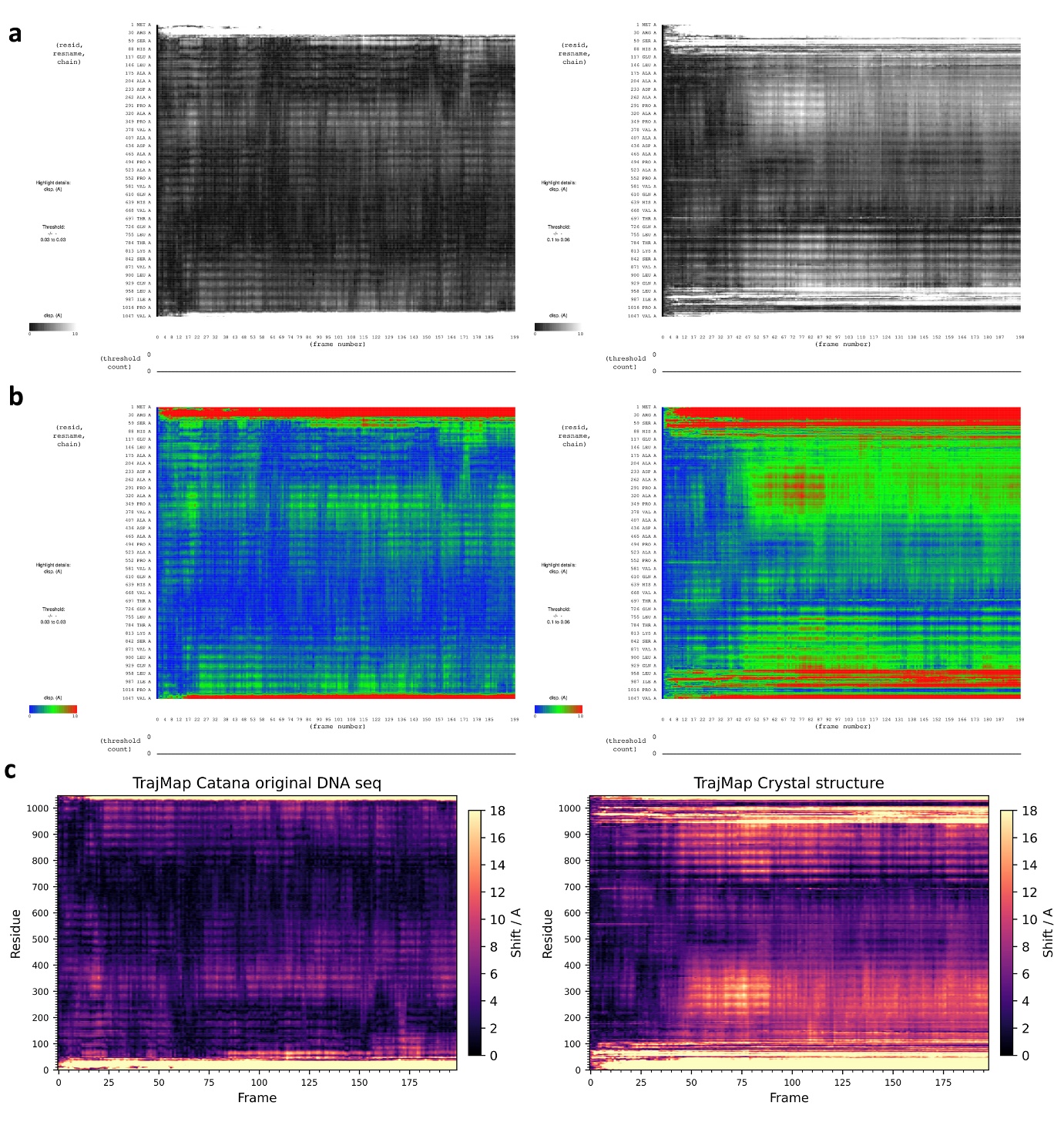


**Figure S2.** A comparison of: a) heatmaps obtained with VMD Timeline tool when plotting “displacement” of aligned trajectories, with a default colormap and range adjusted to match results of trajectory maps, b) heatmaps obtained with VMD Timeline tool after adjusting the colormap to the other available one, c) results of trajectory maps. All three analyses were applied to the identical simulations (Case study 1).


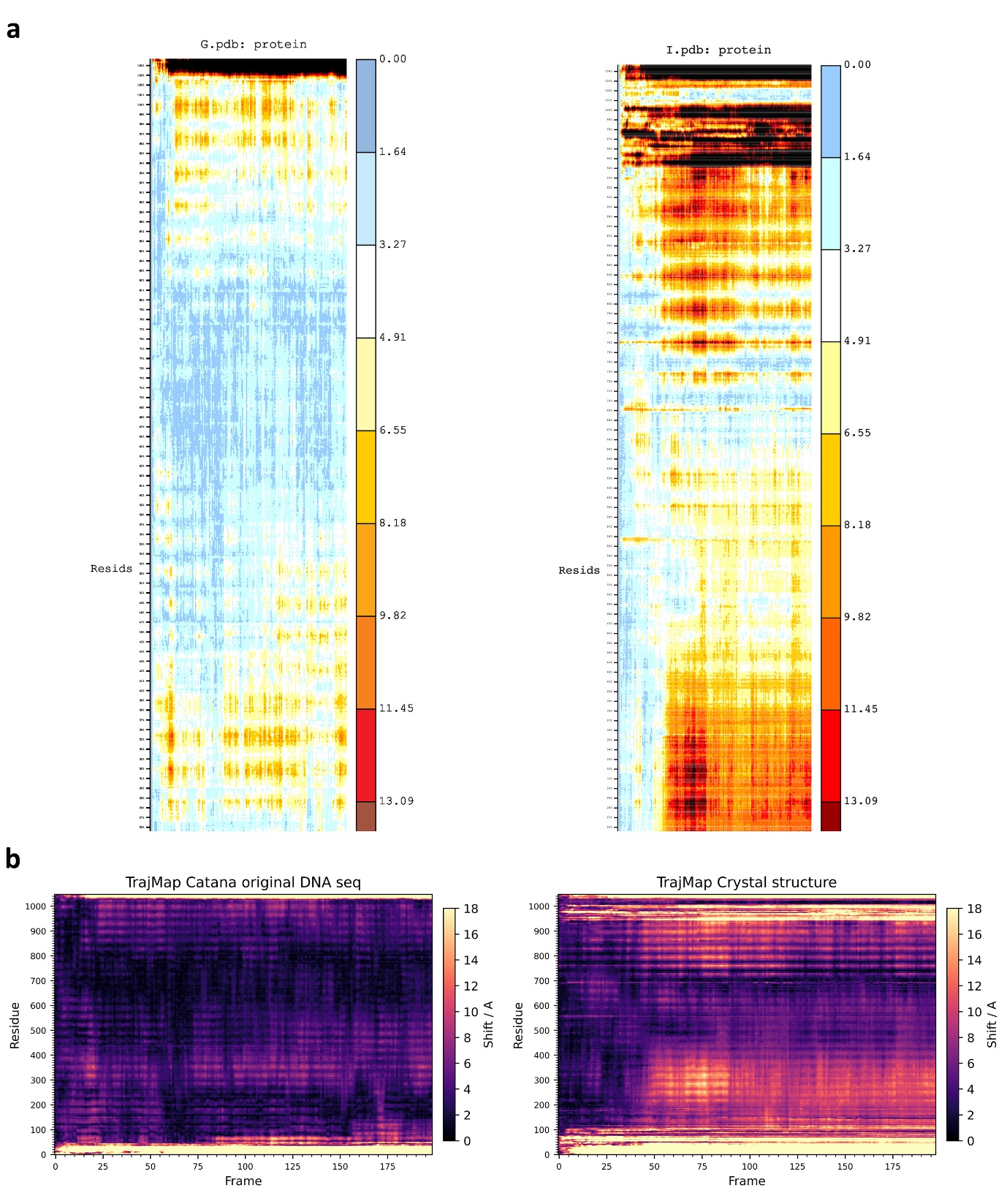


**Figure S3**. A comparison of per-residue RMSD heatmaps obtained with VMD RMSD Visualizer Tool plugin, which visualizes them using VMD Heatmapper tool (panel **a**), compared to trajectory maps of same simulations (panel **b**), (Case study 1). Because of the size (number of residues) of the proteins in simulations, VMD Heatmapper was unable to display the full image or render the heatmap fully, so the rendered image appears to be cut off where the rest of the image should be. As a consequence, the *x* axis that represents time is cut out, as well as a part of the protein. We were unable to obtain uncropped results. In all cases identical simulations of TAL protein in complex with DNA were used.


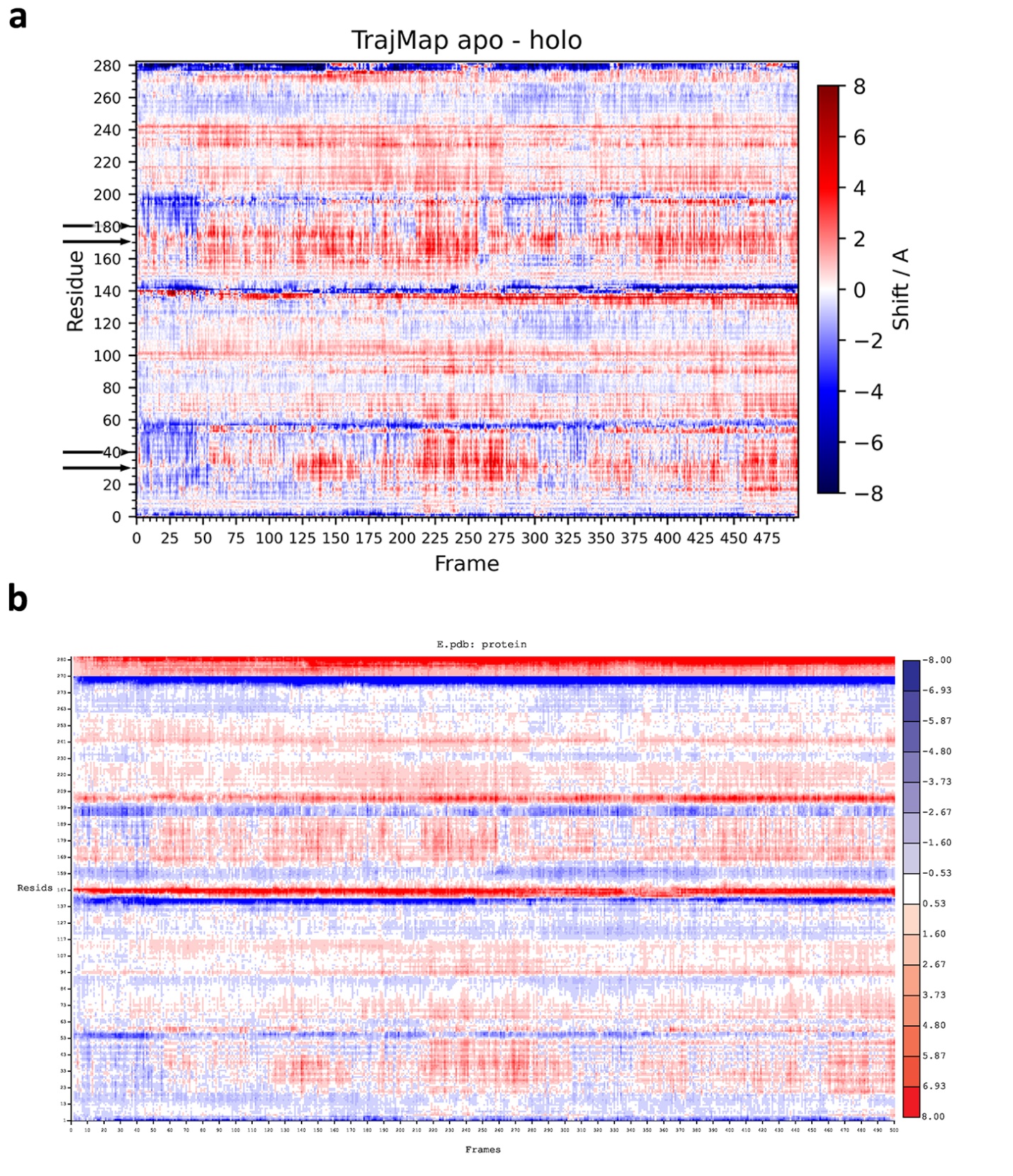


**Figure S4.** A comparison of analysis performed for Case study 2. A difference map of an average of two *apo* simulations and an average of two *holo* simulations (panel **a**), compared to the same difference of averages of per-residue RMSD heatmaps obtained with VMD Heatmapper tool (panel **b**). Range is identical in both heatmaps, from -8 Å to +8 Å. Because VMD tool wasn’t made for this particular purpose, making a difference map using VMD is less straightforward than using trajectory maps tool. Furthermore, that use (which we describe in paper) isn’t mentioned in neither the documentation, nor in literature to the best of our knowledge. As a consequence, an average user would hardly be able to produce these results using VMD. Because of the knowledge that we gained through development of trajectory maps tool, we were able to produce these results with VMD, but it was still demanding and time-consuming task.
